# *catena*-Poly[[[triaqua­europium(III)]-μ-(1*H*-benzimidazole-5,6-dicarboxyl­ato-κ^2^ *O* ^5^:*O* ^6^)-μ-(1*H*,3*H*-benzimidazol-3-ium-5,6-dicarboxyl­ato-κ^3^ *O* ^5^:*O* ^6^,*O* ^6′^)] dihydrate]

**DOI:** 10.1107/S1600536811033496

**Published:** 2011-08-27

**Authors:** Xiao-Ye Chen, Shu-Min Huo, Jing-Jun Lin, Xia Cai, Rong-Hua Zeng

**Affiliations:** a School of Chemistry and the Environment, South China Normal University, Guangzhou 510006, People’s Republic of China; b Key Laboratory of Technology on Electrochemical Energy Storage and Power Generation in Guangdong Universities, South China Normal University, Guangzhou 510006, People’s Republic of China

## Abstract

In the title one-dimensional coordination polymer, {[Eu(C_9_H_4_N_2_O_4_)(C_9_H_5_N_2_O_4_)(H_2_O)_3_]·2H_2_O}_*n*_, one of the 1*H*-benzimidazole-5,6-dicarboxyl­ate (Hbdc) ligands is protonated at the imidazole group (H_2_bdc). The Eu^III^ ion is eight-coordinated by two O atoms from two Hbdc ligands, three O atoms from two H_2_bdc ligands and three water mol­ecules, showing a distorted square-anti­prismatic geometry. The Eu^III^ ions are bridged by the carboxyl­ate groups of the Hbdc and H_2_bdc ligands, forming a chain along [110], with an Eu⋯Eu separation of 5.4594 (3) Å. These chains are further connected by inter­molecular O—H⋯O, N—H⋯O and N—H⋯N hydrogen bonds, as well as π–π inter­actions between the imidazole and benzene rings [centroid–centroid distances = 3.558 (3), 3.906 (2), 3.397 (3), 3.796 (2) and 3.898 (2) Å], into a three-dimensional supra­molecular network.

## Related literature

For background to 1*H*-benzimidazole-5,6-dicarboxyl­ate complexes, see: Fu *et al.* (2009 ▶); Huang *et al.* (2009 ▶); Pan *et al.* (2010 ▶); Wei *et al.* (2009 ▶); Yao *et al.* (2008 ▶).
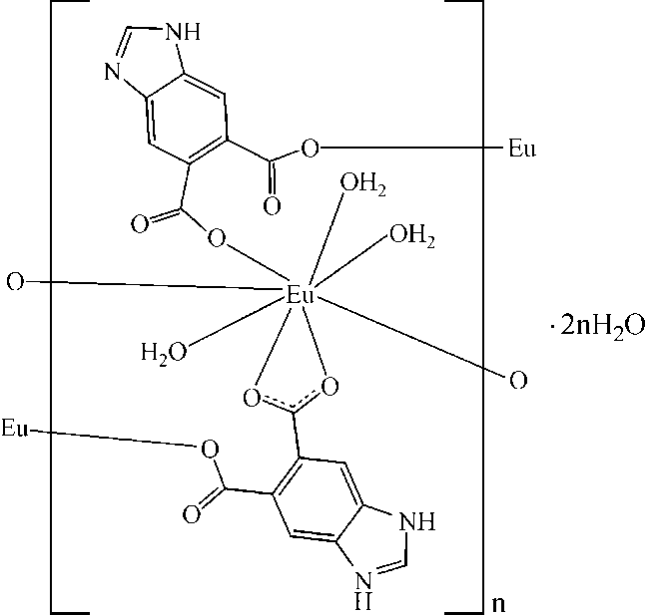


## Experimental

### 

#### Crystal data

[Eu(C_9_H_4_N_2_O_4_)(C_9_H_5_N_2_O_4_)(H_2_O)_3_]·2H_2_O
*M* *_r_* = 651.33
Triclinic, 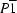

*a* = 8.4530 (4) Å
*b* = 10.9757 (6) Å
*c* = 12.7124 (7) Å
α = 112.112 (1)°
β = 91.614 (1)°
γ = 104.453 (1)°
*V* = 1048.25 (10) Å^3^
*Z* = 2
Mo *K*α radiation
μ = 3.08 mm^−1^
*T* = 298 K
0.24 × 0.22 × 0.20 mm

#### Data collection

Bruker APEXII CCD diffractometer
Absorption correction: multi-scan (*SADABS*; Bruker, 2001 ▶) *T*_min_ = 0.526, *T*_max_ = 0.578
5435 measured reflections
3711 independent reflections
3454 reflections with *I* > 2σ(*I*)
*R*_int_ = 0.016

#### Refinement

*R*[*F* ^2^ > 2σ(*F* ^2^)] = 0.024
*wR*(*F* ^2^) = 0.057
*S* = 1.04
3711 reflections
328 parameters
H atoms treated by a mixture of independent and constrained refinement
Δρ_max_ = 0.63 e Å^−3^
Δρ_min_ = −0.67 e Å^−3^

### 

Data collection: *APEX2* (Bruker, 2007 ▶); cell refinement: *SAINT* (Bruker, 2007 ▶); data reduction: *SAINT*; program(s) used to solve structure: *SHELXS97* (Sheldrick, 2008 ▶); program(s) used to refine structure: *SHELXL97* (Sheldrick, 2008 ▶); molecular graphics: *ORTEPIII* (Burnett & Johnson, 1996 ▶) and *PLATON* (Spek, 2009 ▶); software used to prepare material for publication: *SHELXL97*.

## Supplementary Material

Crystal structure: contains datablock(s) I, glogal. DOI: 10.1107/S1600536811033496/hy2459sup1.cif

Structure factors: contains datablock(s) I. DOI: 10.1107/S1600536811033496/hy2459Isup2.hkl

Additional supplementary materials:  crystallographic information; 3D view; checkCIF report

## Figures and Tables

**Table 1:** Hydrogen-bond geometry (Å, °)

*D*—H⋯*A*	*D*—H	H⋯*A*	*D*⋯*A*	*D*—H⋯*A*
O1*W*—H1*WA*⋯O1	0.85	1.95	2.727 (4)	152
O1*W*—H1*WB*⋯O4^i^	0.85	1.93	2.709 (4)	152
O2*W*—H2*WA*⋯O8	0.85	1.89	2.687 (4)	155
O2*W*—H2*WB*⋯O4*W*	0.85	2.23	2.608 (5)	107
O3*W*—H3*WA*⋯O7^ii^	0.85	2.01	2.815 (4)	159
O3*W*—H3*WB*⋯O5*W*^ii^	0.85	1.96	2.685 (4)	142
O4*W*—H4*WA*⋯O2^iii^	0.85	2.19	2.968 (4)	153
O4*W*—H4*WB*⋯O1*W*^iv^	0.85	2.49	3.022 (4)	122
O4*W*—H4*WB*⋯O4^iii^	0.85	2.37	3.151 (4)	153
O5*W*—H5*WA*⋯O5	0.85	1.97	2.815 (4)	172
O5*W*—H5*WB*⋯O4^v^	0.85	1.99	2.757 (4)	150
N1—H1⋯O1^vi^	0.86	2.06	2.900 (4)	165
N3—H3*A*⋯N2^vii^	0.86	1.88	2.725 (5)	168
N4—H4⋯O6^viii^	0.86	1.98	2.750 (4)	148

Symmetry codes: (i) 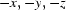; (ii) 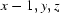; (iii) 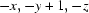; (iv) 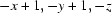; (v) 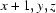; (vi) 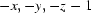; (vii) 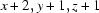; (viii) 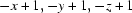.

**Fig. 1.**
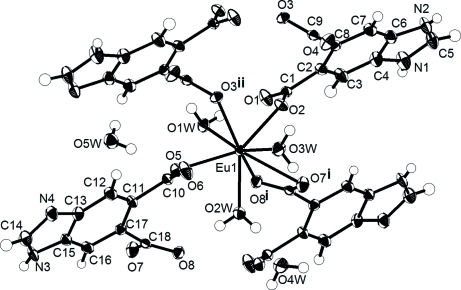
The structure of the title compound, showing displacement ellipsoids drawn at the 30% probability level. [Symmetry codes: (i) 1-x, 1-y, -z; (ii) -x, -y, -z.]

**Fig. 2.**
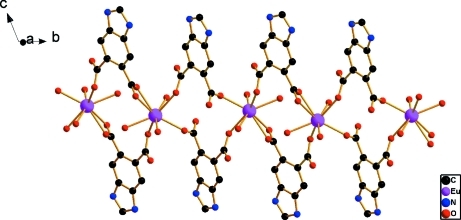
The crystal packing of the title compound, showing the chain structure extending along [1 1 0].

**Fig. 3.**
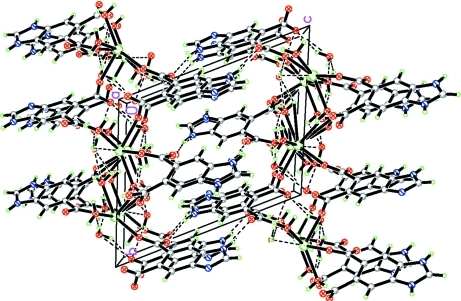
The crystal packing of the title compound, showing the three-dimensional supramolecular network. Hydrogen bonds are shown as dashed lines.

## supplementary crystallographic information

### Comment

There is currently much interest in employing N-heterocyclic carboxylic acids as multidentate ligands to design metal coordination polymers with intriguing structures and potential applications. Particular attention has been paid to 1*H*-benzimidazole-5,6-dicarboxylic acid (H_3_bdc) ligand. It has rich coordination sites (two N atoms and four O atoms) and can be partially or fully deprotonated to produce [H_2_bdc]^-^, [Hbdc]^2-^ and [bdc]^3-^ anions at different pH values. Thus, H_3_bdc can potentially afford different coordination modes in multicoordinated ways with transition metal ions (Fu *et al.*, 2009; Wei *et al.*, 2009) or rare earth metal ions (Huang *et al.*, 2009; Pan *et al.*, 2010; Yao *et al.*, 2008) to form metal coordination polymers with various structures and interesting properties. In this paper, we report the crystal structure of the title compound, which was synthesized under hydrothermal conditions.

As shown in Fig. 1, the title compound has two forms of the ligands, [Hbdc]^2-^ and [H_2_bdc]^-^ anions, and the latter is protonated at the imidazole group. The Eu^III^ ion is eight-coordinated by five O atoms from two Hbdc and two H_2_bdc ligands and by three water molecules. The coordination geometry around the Eu^III^ ion can be described as distorted square-antiprismatic, with Eu—O bond lengths ranging from 2.343 (2) to 2.656 (3) Å and O—Eu—O bond angles varying from 68.99 (9) to 156.77 (9)°. In the crystal, the Eu^III^ ions are alternately bridged by the carboxylate groups of the Hbdc and H_2_bdc ligands, forming chains along [1 1 0] (Fig. 2). These chains are further connected by intermolecular O—H···O, N—H···O and N—H···N hydrogen bonds (Table 1), as well as π–π interactions between the imidazole and benzene rings [centroid–centroid distances = 3.558 (3), 3.906 (2), 3.397 (3), 3.796 (2) and 3.898 (2) Å], into a three-dimensional supramolecular network (Fig. 3).

### Experimental

A mixture of Eu_2_O_3_ (0.352 g, 1 mmol), H_3_bdc (0.206 g, 1 mmol), water (10 ml) in the presence of HClO_4_ (0.039 g, 0.385 mmol) was stirred vigorously for 30 min and then sealed in a 20 ml Teflon-lined stainless-steel autoclave. The autoclave was heated and maintained at 443 K for 3 days, and then cooled to room temperature at 5K h^-1^. Colorless block crystals of the title compound were obtained.

### Refinement

Water H atoms were tentatively located in difference Fourier maps and were refined with distance restraints of O–H = 0.85 and H···H = 1.35 Å and with *U*_iso_(H) = 1.5*U*_eq_(O). H atoms of the ligands were positioned geometrically and refined as riding atoms, with C—H = 0.93 and N—H = 0.86 Å and with *U*_iso_(H) = 1.2*U*_eq_(C, N).

### Crystal data

**Table:**

[Eu(C_9_H_4_N_2_O_4_)(C_9_H_5_N_2_O_4_)(H_2_O)_3_]·2H_2_O	*Z* = 2
*M**_r_* = 651.33	*F*(000) = 644
Triclinic, *P*1	*D*_x_ = 2.064 Mg m^−^^3^
Hall symbol: -P 1	Mo *K*α radiation, λ = 0.71073 Å
*a* = 8.4530 (4) Å	Cell parameters from 3764 reflections
*b* = 10.9757 (6) Å	θ = 2.8–25.2°
*c* = 12.7124 (7) Å	µ = 3.08 mm^−^^1^
α = 112.112 (1)°	*T* = 298 K
β = 91.614 (1)°	Block, colorless
γ = 104.453 (1)°	0.24 × 0.22 × 0.20 mm
*V* = 1048.25 (10) Å^3^	

### Data collection

**Table:**

Bruker APEXII CCD diffractometer	3711 independent reflections
Radiation source: fine-focus sealed tube	3454 reflections with *I* > 2σ(*I*)
graphite	*R*_int_ = 0.016
φ and ω scans	θ_max_ = 25.2°, θ_min_ = 1.8°
Absorption correction: multi-scan (*SADABS*; Bruker, 2001)	*h* = −10→10
*T*_min_ = 0.526, *T*_max_ = 0.578	*k* = −13→7
5435 measured reflections	*l* = −13→15

### Refinement

**Table:**

Refinement on *F*^2^	Primary atom site location: structure-invariant direct methods
Least-squares matrix: full	Secondary atom site location: difference Fourier map
*R*[*F*^2^ > 2σ(*F*^2^)] = 0.024	Hydrogen site location: inferred from neighbouring sites
*wR*(*F*^2^) = 0.057	H atoms treated by a mixture of independent and constrained refinement
*S* = 1.04	*w* = 1/[σ^2^(*F*_o_^2^) + (0.0269*P*)^2^ + 1.5288*P*] where *P* = (*F*_o_^2^ + 2*F*_c_^2^)/3
3711 reflections	(Δ/σ)_max_ = 0.001
328 parameters	Δρ_max_ = 0.63 e Å^−^^3^
0 restraints	Δρ_min_ = −0.67 e Å^−^^3^

### Fractional atomic coordinates and isotropic or equivalent isotropic displacement parameters (Å^2^)

**Table:**

	*x*	*y*	*z*	*U*_iso_*/*U*_eq_	
C1	0.0343 (4)	0.0408 (4)	−0.2244 (3)	0.0189 (8)	
C2	−0.1313 (4)	−0.0377 (4)	−0.2964 (3)	0.0187 (8)	
C3	−0.1457 (5)	−0.0513 (4)	−0.4085 (3)	0.0244 (9)	
H3	−0.051 (5)	−0.019 (4)	−0.434 (3)	0.029*	
C4	−0.3019 (5)	−0.1056 (4)	−0.4725 (3)	0.0232 (8)	
C5	−0.5240 (5)	−0.1795 (4)	−0.5977 (3)	0.0299 (9)	
H5	−0.5915	−0.2033	−0.6655	0.036*	
C6	−0.4412 (4)	−0.1460 (4)	−0.4248 (3)	0.0213 (8)	
C7	−0.4269 (4)	−0.1359 (4)	−0.3119 (3)	0.0210 (8)	
H7	−0.5196	−0.1642	−0.2801	0.025*	
C8	−0.2722 (4)	−0.0831 (4)	−0.2488 (3)	0.0171 (7)	
C9	−0.2606 (4)	−0.0819 (4)	−0.1297 (3)	0.0185 (8)	
C10	0.5024 (4)	0.4467 (4)	0.2501 (3)	0.0198 (8)	
C11	0.6702 (4)	0.5243 (4)	0.3216 (3)	0.0174 (7)	
C12	0.6891 (4)	0.5302 (4)	0.4319 (3)	0.0219 (8)	
H12	0.6019	0.4883	0.4609	0.026*	
C13	0.8420 (4)	0.6003 (4)	0.4979 (3)	0.0198 (8)	
C14	1.0585 (5)	0.7001 (4)	0.6311 (3)	0.0271 (9)	
H14	1.1247	0.7334	0.7012	0.033*	
C15	0.9760 (4)	0.6562 (4)	0.4530 (3)	0.0190 (8)	
C16	0.9601 (4)	0.6502 (4)	0.3422 (3)	0.0198 (8)	
H16	1.0501	0.6869	0.3122	0.024*	
C17	0.8053 (4)	0.5876 (4)	0.2777 (3)	0.0174 (7)	
C18	0.7878 (4)	0.6050 (4)	0.1667 (3)	0.0174 (7)	
Eu1	0.24165 (2)	0.308010 (18)	0.021383 (14)	0.01508 (7)	
N1	−0.3591 (4)	−0.1283 (4)	−0.5833 (3)	0.0302 (8)	
H1	−0.3003	−0.1126	−0.6334	0.036*	
N2	−0.5797 (4)	−0.1922 (4)	−0.5056 (3)	0.0271 (8)	
N3	1.1094 (4)	0.7157 (3)	0.5386 (3)	0.0239 (7)	
H3A	1.2081	0.7556	0.5326	0.029*	
N4	0.8992 (4)	0.6303 (3)	0.6108 (3)	0.0248 (7)	
H4	0.8422	0.6080	0.6591	0.030*	
O1	0.1619 (3)	0.0246 (3)	−0.2694 (2)	0.0282 (6)	
O2	0.0329 (3)	0.1254 (3)	−0.1235 (2)	0.0218 (6)	
O3	−0.1766 (3)	−0.1539 (3)	−0.1112 (2)	0.0232 (6)	
O4	−0.3436 (3)	−0.0182 (3)	−0.0613 (2)	0.0286 (6)	
O5	0.5008 (3)	0.3761 (3)	0.1434 (2)	0.0233 (6)	
O6	0.3781 (3)	0.4550 (3)	0.2988 (3)	0.0357 (7)	
O7	0.8941 (3)	0.5849 (3)	0.1007 (2)	0.0251 (6)	
O8	0.6729 (3)	0.6529 (3)	0.1488 (2)	0.0217 (6)	
O1W	0.3986 (3)	0.1495 (3)	−0.0826 (2)	0.0225 (6)	
H1WA	0.3469	0.0936	−0.1481	0.027*	
H1WB	0.4122	0.1032	−0.0442	0.027*	
O2W	0.3436 (3)	0.5492 (3)	0.1002 (3)	0.0327 (7)	
H2WA	0.4404	0.5907	0.1361	0.039*	
H2WB	0.3295	0.5815	0.0506	0.039*	
O3W	0.0181 (3)	0.3743 (3)	0.1123 (2)	0.0277 (6)	
H3WA	−0.0036	0.4498	0.1257	0.033*	
H3WB	−0.0456	0.3322	0.1459	0.033*	
O4W	0.2568 (4)	0.7730 (3)	0.1413 (3)	0.0389 (7)	
H4WA	0.1577	0.7772	0.1398	0.047*	
H4WB	0.3140	0.8382	0.1259	0.047*	
O5W	0.7262 (3)	0.2225 (3)	0.1315 (2)	0.0302 (6)	
H5WA	0.6621	0.2706	0.1300	0.036*	
H5WB	0.7262	0.1686	0.0628	0.036*	

### Atomic displacement parameters (Å^2^)

**Table:**

	*U*^11^	*U*^22^	*U*^33^	*U*^12^	*U*^13^	*U*^23^
C1	0.0164 (18)	0.0202 (19)	0.0207 (19)	−0.0007 (15)	−0.0012 (14)	0.0129 (16)
C2	0.0184 (18)	0.0187 (19)	0.0173 (18)	0.0028 (15)	0.0028 (14)	0.0067 (15)
C3	0.0187 (19)	0.033 (2)	0.0191 (19)	0.0015 (17)	0.0035 (15)	0.0109 (17)
C4	0.0226 (19)	0.032 (2)	0.0153 (18)	0.0067 (17)	0.0029 (15)	0.0101 (16)
C5	0.025 (2)	0.041 (3)	0.019 (2)	0.0059 (18)	−0.0054 (16)	0.0098 (18)
C6	0.0217 (19)	0.022 (2)	0.0167 (18)	0.0034 (16)	−0.0014 (14)	0.0063 (15)
C7	0.0173 (18)	0.025 (2)	0.0215 (19)	0.0038 (16)	0.0048 (15)	0.0121 (16)
C8	0.0197 (18)	0.0151 (18)	0.0170 (18)	0.0041 (14)	0.0004 (14)	0.0077 (15)
C9	0.0188 (18)	0.0167 (18)	0.0170 (18)	−0.0018 (15)	−0.0015 (14)	0.0080 (15)
C10	0.0157 (18)	0.0199 (19)	0.023 (2)	0.0002 (15)	−0.0022 (15)	0.0109 (16)
C11	0.0139 (17)	0.0192 (19)	0.0152 (17)	0.0018 (14)	−0.0015 (13)	0.0046 (15)
C12	0.0168 (18)	0.026 (2)	0.023 (2)	0.0023 (16)	0.0042 (15)	0.0119 (17)
C13	0.0201 (18)	0.025 (2)	0.0155 (18)	0.0077 (16)	0.0029 (14)	0.0082 (16)
C14	0.029 (2)	0.028 (2)	0.018 (2)	0.0055 (18)	−0.0055 (16)	0.0060 (17)
C15	0.0159 (17)	0.0169 (19)	0.0201 (19)	0.0034 (15)	−0.0010 (14)	0.0038 (15)
C16	0.0170 (18)	0.0217 (19)	0.0204 (19)	0.0017 (15)	0.0030 (14)	0.0102 (16)
C17	0.0189 (18)	0.0171 (18)	0.0168 (18)	0.0042 (15)	0.0017 (14)	0.0080 (15)
C18	0.0144 (17)	0.0149 (18)	0.0190 (18)	−0.0025 (14)	−0.0023 (14)	0.0071 (15)
Eu1	0.01417 (10)	0.01681 (11)	0.01459 (10)	0.00295 (7)	0.00063 (6)	0.00757 (7)
N1	0.0256 (18)	0.049 (2)	0.0148 (16)	0.0055 (16)	0.0020 (13)	0.0141 (16)
N2	0.0207 (16)	0.035 (2)	0.0213 (17)	0.0029 (15)	−0.0044 (13)	0.0102 (15)
N3	0.0139 (15)	0.0304 (19)	0.0211 (17)	0.0001 (13)	−0.0064 (12)	0.0079 (14)
N4	0.0252 (17)	0.0329 (19)	0.0171 (16)	0.0051 (15)	0.0009 (13)	0.0129 (15)
O1	0.0166 (13)	0.0372 (17)	0.0244 (14)	0.0037 (12)	0.0034 (11)	0.0075 (13)
O2	0.0195 (13)	0.0199 (14)	0.0184 (13)	−0.0001 (11)	−0.0002 (10)	0.0032 (11)
O3	0.0231 (14)	0.0250 (14)	0.0281 (14)	0.0046 (11)	0.0029 (11)	0.0191 (12)
O4	0.0384 (16)	0.0338 (16)	0.0204 (14)	0.0179 (13)	0.0094 (12)	0.0128 (12)
O5	0.0226 (13)	0.0224 (14)	0.0220 (14)	0.0039 (11)	−0.0043 (11)	0.0077 (12)
O6	0.0157 (14)	0.052 (2)	0.0389 (17)	0.0044 (13)	0.0067 (12)	0.0197 (15)
O7	0.0228 (14)	0.0356 (16)	0.0224 (14)	0.0111 (12)	0.0055 (11)	0.0155 (12)
O8	0.0192 (13)	0.0260 (14)	0.0260 (14)	0.0080 (11)	0.0040 (10)	0.0160 (12)
O1W	0.0239 (14)	0.0249 (14)	0.0194 (13)	0.0065 (11)	0.0000 (10)	0.0099 (11)
O2W	0.0279 (15)	0.0213 (15)	0.0426 (18)	0.0016 (12)	−0.0120 (13)	0.0103 (13)
O3W	0.0262 (14)	0.0305 (16)	0.0355 (16)	0.0138 (12)	0.0128 (12)	0.0187 (13)
O4W	0.0299 (16)	0.0295 (17)	0.061 (2)	0.0109 (13)	0.0063 (14)	0.0200 (15)
O5W	0.0301 (15)	0.0324 (16)	0.0254 (15)	0.0093 (13)	0.0014 (12)	0.0084 (13)

### Geometric parameters (Å, °)

**Table:**

C1—O1	1.256 (4)	C14—H14	0.9300
C1—O2	1.272 (4)	C15—N3	1.382 (4)
C1—C2	1.507 (5)	C15—C16	1.386 (5)
C2—C3	1.374 (5)	C16—C17	1.387 (5)
C2—C8	1.416 (5)	C16—H16	0.9300
C3—C4	1.394 (5)	C17—C18	1.502 (5)
C3—H3	0.91 (4)	C18—O7	1.249 (4)
C4—N1	1.385 (5)	C18—O8	1.267 (4)
C4—C6	1.391 (5)	Eu1—O3^i^	2.344 (2)
C5—N2	1.318 (5)	Eu1—O2W	2.360 (3)
C5—N1	1.345 (5)	Eu1—O3W	2.369 (3)
C5—H5	0.9300	Eu1—O2	2.407 (2)
C6—N2	1.388 (5)	Eu1—O5	2.425 (2)
C6—C7	1.396 (5)	Eu1—O8^ii^	2.453 (2)
C7—C8	1.379 (5)	Eu1—O1W	2.460 (2)
C7—H7	0.9300	Eu1—O7^ii^	2.656 (3)
C8—C9	1.509 (5)	N1—H1	0.8600
C9—O4	1.248 (4)	N3—H3A	0.8600
C9—O3	1.263 (4)	N4—H4	0.8600
C10—O6	1.239 (4)	O1W—H1WA	0.8500
C10—O5	1.282 (4)	O1W—H1WB	0.8500
C10—C11	1.518 (5)	O2W—H2WA	0.8500
C11—C12	1.382 (5)	O2W—H2WB	0.8498
C11—C17	1.424 (5)	O3W—H3WA	0.8500
C12—C13	1.387 (5)	O3W—H3WB	0.8500
C12—H12	0.9300	O4W—H4WA	0.8500
C13—N4	1.388 (5)	O4W—H4WB	0.8500
C13—C15	1.393 (5)	O5W—H5WA	0.8499
C14—N3	1.319 (5)	O5W—H5WB	0.8500
C14—N4	1.335 (5)		
			
O1—C1—O2	125.0 (3)	O2W—Eu1—O5	70.78 (9)
O1—C1—C2	118.5 (3)	O3W—Eu1—O5	116.75 (9)
O2—C1—C2	116.3 (3)	O2—Eu1—O5	147.35 (9)
C3—C2—C8	120.3 (3)	O3^i^—Eu1—O8^ii^	147.47 (9)
C3—C2—C1	117.4 (3)	O2W—Eu1—O8^ii^	79.57 (10)
C8—C2—C1	122.0 (3)	O3W—Eu1—O8^ii^	123.60 (9)
C2—C3—C4	118.8 (3)	O2—Eu1—O8^ii^	80.30 (8)
C2—C3—H3	116 (3)	O5—Eu1—O8^ii^	103.24 (8)
C4—C3—H3	125 (3)	O3^i^—Eu1—O1W	81.33 (9)
N1—C4—C6	105.6 (3)	O2W—Eu1—O1W	124.88 (9)
N1—C4—C3	133.3 (3)	O3W—Eu1—O1W	156.77 (9)
C6—C4—C3	121.0 (3)	O2—Eu1—O1W	77.56 (8)
N2—C5—N1	112.8 (3)	O5—Eu1—O1W	73.73 (8)
N2—C5—H5	123.6	O8^ii^—Eu1—O1W	69.16 (8)
N1—C5—H5	123.6	O3^i^—Eu1—O7^ii^	142.44 (8)
N2—C6—C4	109.1 (3)	O2W—Eu1—O7^ii^	68.99 (9)
N2—C6—C7	130.4 (3)	O3W—Eu1—O7^ii^	72.82 (8)
C4—C6—C7	120.5 (3)	O2—Eu1—O7^ii^	71.98 (9)
C8—C7—C6	118.5 (3)	O5—Eu1—O7^ii^	135.26 (8)
C8—C7—H7	120.8	O8^ii^—Eu1—O7^ii^	50.84 (8)
C6—C7—H7	120.8	O1W—Eu1—O7^ii^	115.56 (8)
C7—C8—C2	120.9 (3)	O3^i^—Eu1—C18^ii^	154.10 (9)
C7—C8—C9	116.8 (3)	O2W—Eu1—C18^ii^	73.39 (10)
C2—C8—C9	122.2 (3)	O3W—Eu1—C18^ii^	98.19 (10)
O4—C9—O3	125.9 (3)	O2—Eu1—C18^ii^	73.86 (9)
O4—C9—C8	117.4 (3)	O5—Eu1—C18^ii^	121.97 (9)
O3—C9—C8	116.5 (3)	O8^ii^—Eu1—C18^ii^	25.48 (9)
O6—C10—O5	125.0 (3)	O1W—Eu1—C18^ii^	92.14 (9)
O6—C10—C11	118.1 (3)	O7^ii^—Eu1—C18^ii^	25.38 (9)
O5—C10—C11	116.9 (3)	O3^i^—Eu1—H2WB	146.1
C12—C11—C17	120.4 (3)	O2W—Eu1—H2WB	16.6
C12—C11—C10	117.4 (3)	O3W—Eu1—H2WB	80.6
C17—C11—C10	122.2 (3)	O2—Eu1—H2WB	125.6
C11—C12—C13	118.0 (3)	O5—Eu1—H2WB	83.6
C11—C12—H12	121.0	O8^ii^—Eu1—H2WB	65.6
C13—C12—H12	121.0	O1W—Eu1—H2WB	122.2
C12—C13—N4	132.5 (3)	O7^ii^—Eu1—H2WB	53.7
C12—C13—C15	121.4 (3)	C18^ii^—Eu1—H2WB	56.9
N4—C13—C15	106.1 (3)	C5—N1—C4	107.0 (3)
N3—C14—N4	111.0 (3)	C5—N1—H1	126.5
N3—C14—H14	124.5	C4—N1—H1	126.5
N4—C14—H14	124.5	C5—N2—C6	105.5 (3)
N3—C15—C16	131.3 (3)	C14—N3—C15	107.8 (3)
N3—C15—C13	107.3 (3)	C14—N3—H3A	126.1
C16—C15—C13	121.4 (3)	C15—N3—H3A	126.1
C15—C16—C17	117.6 (3)	C14—N4—C13	107.8 (3)
C15—C16—H16	121.2	C14—N4—H4	126.1
C17—C16—H16	121.2	C13—N4—H4	126.1
C16—C17—C11	121.0 (3)	C1—O2—Eu1	133.9 (2)
C16—C17—C18	115.4 (3)	C9—O3—Eu1^i^	129.4 (2)
C11—C17—C18	123.3 (3)	C10—O5—Eu1	116.1 (2)
O7—C18—O8	122.0 (3)	C18—O7—Eu1^ii^	88.9 (2)
O7—C18—C17	119.8 (3)	C18—O8—Eu1^ii^	98.1 (2)
O8—C18—C17	117.8 (3)	Eu1—O1W—H1WA	111.5
O7—C18—Eu1^ii^	65.67 (19)	Eu1—O1W—H1WB	108.5
O8—C18—Eu1^ii^	56.45 (17)	H1WA—O1W—H1WB	107.7
C17—C18—Eu1^ii^	169.2 (2)	Eu1—O2W—H2WA	122.2
O3^i^—Eu1—O2W	130.52 (10)	Eu1—O2W—H2WB	111.0
O3^i^—Eu1—O3W	80.33 (9)	H2WA—O2W—H2WB	107.7
O2W—Eu1—O3W	78.17 (10)	Eu1—O3W—H3WA	125.9
O3^i^—Eu1—O2	80.26 (9)	Eu1—O3W—H3WB	125.9
O2W—Eu1—O2	140.57 (9)	H3WA—O3W—H3WB	107.7
O3W—Eu1—O2	85.39 (9)	H4WA—O4W—H4WB	107.7
O3^i^—Eu1—O5	80.37 (9)	H5WA—O5W—H5WB	107.7
			
O1—C1—C2—C3	−39.6 (5)	C16—C17—C18—O7	47.4 (5)
O2—C1—C2—C3	136.4 (4)	C11—C17—C18—O7	−139.2 (4)
O1—C1—C2—C8	147.3 (4)	C16—C17—C18—O8	−125.8 (4)
O2—C1—C2—C8	−36.7 (5)	C11—C17—C18—O8	47.7 (5)
C8—C2—C3—C4	2.4 (6)	C16—C17—C18—Eu1^ii^	−70.7 (14)
C1—C2—C3—C4	−170.8 (4)	C11—C17—C18—Eu1^ii^	102.8 (12)
C2—C3—C4—N1	177.5 (4)	N2—C5—N1—C4	0.0 (5)
C2—C3—C4—C6	−0.2 (6)	C6—C4—N1—C5	0.0 (5)
N1—C4—C6—N2	0.0 (4)	C3—C4—N1—C5	−178.0 (5)
C3—C4—C6—N2	178.3 (4)	N1—C5—N2—C6	0.0 (5)
N1—C4—C6—C7	−179.8 (4)	C4—C6—N2—C5	0.0 (5)
C3—C4—C6—C7	−1.5 (6)	C7—C6—N2—C5	179.7 (4)
N2—C6—C7—C8	−178.7 (4)	N4—C14—N3—C15	1.6 (5)
C4—C6—C7—C8	1.0 (6)	C16—C15—N3—C14	176.2 (4)
C6—C7—C8—C2	1.1 (5)	C13—C15—N3—C14	−1.6 (4)
C6—C7—C8—C9	−176.4 (3)	N3—C14—N4—C13	−1.0 (5)
C3—C2—C8—C7	−2.9 (6)	C12—C13—N4—C14	179.4 (4)
C1—C2—C8—C7	170.0 (3)	C15—C13—N4—C14	0.0 (4)
C3—C2—C8—C9	174.5 (4)	O1—C1—O2—Eu1	8.7 (6)
C1—C2—C8—C9	−12.6 (5)	C2—C1—O2—Eu1	−166.9 (2)
C7—C8—C9—O4	−58.4 (5)	O3^i^—Eu1—O2—C1	−112.6 (3)
C2—C8—C9—O4	124.1 (4)	O2W—Eu1—O2—C1	101.4 (3)
C7—C8—C9—O3	116.7 (4)	O3W—Eu1—O2—C1	166.5 (3)
C2—C8—C9—O3	−60.7 (5)	O5—Eu1—O2—C1	−58.2 (4)
O6—C10—C11—C12	37.3 (5)	O8^ii^—Eu1—O2—C1	41.2 (3)
O5—C10—C11—C12	−142.0 (4)	O1W—Eu1—O2—C1	−29.4 (3)
O6—C10—C11—C17	−143.7 (4)	O7^ii^—Eu1—O2—C1	93.1 (3)
O5—C10—C11—C17	37.0 (5)	C18^ii^—Eu1—O2—C1	66.6 (3)
C17—C11—C12—C13	0.7 (6)	O4—C9—O3—Eu1^i^	41.5 (5)
C10—C11—C12—C13	179.8 (3)	C8—C9—O3—Eu1^i^	−133.1 (3)
C11—C12—C13—N4	176.6 (4)	O6—C10—O5—Eu1	17.8 (5)
C11—C12—C13—C15	−4.0 (6)	C11—C10—O5—Eu1	−163.0 (2)
C12—C13—C15—N3	−178.6 (3)	O3^i^—Eu1—O5—C10	−75.4 (2)
N4—C13—C15—N3	1.0 (4)	O2W—Eu1—O5—C10	63.8 (2)
C12—C13—C15—C16	3.4 (6)	O3W—Eu1—O5—C10	−1.4 (3)
N4—C13—C15—C16	−177.1 (3)	O2—Eu1—O5—C10	−129.7 (2)
N3—C15—C16—C17	−176.9 (4)	O8^ii^—Eu1—O5—C10	137.6 (2)
C13—C15—C16—C17	0.6 (5)	O1W—Eu1—O5—C10	−159.1 (3)
C15—C16—C17—C11	−3.8 (5)	O7^ii^—Eu1—O5—C10	90.8 (3)
C15—C16—C17—C18	169.9 (3)	C18^ii^—Eu1—O5—C10	118.8 (2)
C12—C11—C17—C16	3.2 (6)	O8—C18—O7—Eu1^ii^	3.3 (3)
C10—C11—C17—C16	−175.8 (3)	C17—C18—O7—Eu1^ii^	−169.6 (3)
C12—C11—C17—C18	−169.9 (3)	O7—C18—O8—Eu1^ii^	−3.6 (4)
C10—C11—C17—C18	11.1 (5)	C17—C18—O8—Eu1^ii^	169.4 (3)

Symmetry codes: (i) −*x*, −*y*, −*z*; (ii) −*x*+1, −*y*+1, −*z*.

### Hydrogen-bond geometry (Å, °)

**Table:**

*D*—H···*A*	*D*—H	H···*A*	*D*···*A*	*D*—H···*A*
O1W—H1WA···O1	0.85	1.95	2.727 (4)	152
O1W—H1WB···O4^i^	0.85	1.93	2.709 (4)	152
O2W—H2WA···O8	0.85	1.89	2.687 (4)	155
O2W—H2WB···O4W	0.85	2.23	2.608 (5)	107
O3W—H3WA···O7^iii^	0.85	2.01	2.815 (4)	159
O3W—H3WB···O5W^iii^	0.85	1.96	2.685 (4)	142
O4W—H4WA···O2^iv^	0.85	2.19	2.968 (4)	153
O4W—H4WB···O1W^ii^	0.85	2.49	3.022 (4)	122
O4W—H4WB···O4^iv^	0.85	2.37	3.151 (4)	153
O5W—H5WA···O5	0.85	1.97	2.815 (4)	172
O5W—H5WB···O4^v^	0.85	1.99	2.757 (4)	150
N1—H1···O1^vi^	0.86	2.06	2.900 (4)	165
N3—H3A···N2^vii^	0.86	1.88	2.725 (5)	168
N4—H4···O6^viii^	0.86	1.98	2.750 (4)	148

Symmetry codes: (i) −*x*, −*y*, −*z*; (iii) *x*−1, *y*, *z*; (iv) −*x*, −*y*+1, −*z*; (ii) −*x*+1, −*y*+1, −*z*; (v) *x*+1, *y*, *z*; (vi) −*x*, −*y*, −*z*−1; (vii) *x*+2, *y*+1, *z*+1; (viii) −*x*+1, −*y*+1, −*z*+1.
